# Inhibition of Classical and Alternative Complement Pathway by Ravulizumab and Eculizumab

**DOI:** 10.1002/acn3.70251

**Published:** 2025-11-19

**Authors:** Lea Gerischer, Frauke Stascheit, Maximilian Mönch, Paolo Doksani, Carla Dusemund, Meret Herdick, Philipp Mergenthaler, Maike Stein, Amani Suboh, Jutta Schröder‐Braunstein, Guido Wabnitz, Jan D. Lünemann, Sophie Lehnerer, Sarah Hoffmann, Andreas Meisel

**Affiliations:** ^1^ Department of Neurology With Experimental Neurology and Integrated Myasthenia Center Charité—Universitätsmedizin Berlin, Corporate Member of Freie Universität Berlin and Humboldt‐Universität Zu Berlin Berlin Germany; ^2^ Neuroscience Clinical Research Center Charité—Universitätsmedizin Berlin, Corporate Member of Freie Universität Berlin and Humboldt‐Universität Zu Berlin Berlin Germany; ^3^ Institute of Biometry and Clinical Epidemiology Charité—Universitätsmedizin Berlin, Corporate Member of Freie Universität Berlin and Humboldt‐Universität Zu Berlin Berlin Germany; ^4^ Center for Stroke Research Berlin Charité—Universitätsmedizin Berlin Berlin Germany; ^5^ Radcliffe Department of Medicine University of Oxford Oxford UK; ^6^ Laboratory of Complement Diagnostics, Institute of Immunology University Hospital Heidelberg Heidelberg Germany; ^7^ Department of Neurology University Hospital Münster Münster Germany; ^8^ Berlin Institute of Health at Charité—Universitätsmedizin Berlin Digital Health Center Berlin Germany

**Keywords:** complement inhibition, complement pathway, eculizumab, myasthenia gravis, ravulizumab

## Abstract

**Objective:**

To explore the feasibility of classical (CH50) and alternative (AH50) complement pathway activity as potential biomarkers for treatment guidance and monitoring during therapy with ravulizumab in patients with generalized myasthenia gravis (gMG) and compare these to therapeutic drug monitoring under eculizumab.

**Methods:**

In this prospective, exploratory real‐world study CH50 and AH50, eculizumab and ravulizumab blood levels were assessed in patients with acetylcholine‐receptor‐antibody (AChR‐ab) positive gMG. Patients were either pretreated with eculizumab or C5‐inhibitor naïve. Samples were collected before the next infusion (end‐of‐dose). Laboratory data were correlated with patient‐reported subjective duration of the ravulizumab effect.

**Results:**

Overall, 61 patients were enrolled. At the end of their respective dosing interval, median AH50 levels were more strongly suppressed with eculizumab than with ravulizumab (1% [0%–2%] versus 5% [3%–9%]; Cohen's *d* 2.2 [95% CI 1.5–2.8]). Patients who discontinued ravulizumab due to insufficient effects (*n* = 19; 33%) had higher median AH50 levels than those who continued (7% [4%–13%] versus 5% [3%–8%]; Cohen's *d* −0.9 [95% CI −1.3 to −0.4]). In 81% (*n* = 46) of patients, the therapeutic effect of ravulizumab diminished before the subsequent infusion after the standard 8‐week interval. Higher AH50 levels were correlated with earlier symptom recurrence.

**Interpretation:**

Our results indicate potential differences in the ability of eculizumab and ravulizumab to suppress complement pathway activity through their respective dosing intervals. Additionally, higher AH50 levels might be a potential biomarker to predict poor therapy response and faster wearing off of ravulizumab's effect. However, these findings need to be validated in large multicenter studies.

## 1 Introduction

The monoclonal antibody eculizumab was the first complement component 5 (C5) inhibitor that gained regulatory approval for the treatment of patients with acetylcholine‐receptor‐antibody (AChR‐ab) positive, treatment‐refractory generalized myasthenia gravis (gMG). The approval was based on the REGAIN trial results [1]. Ravulizumab has been engineered as a successor to eculizumab by introducing changes to the structure in order to achieve a prolonged half‐life. The prolonged half‐life is achieved through enhanced FcRn binding and recycling of the drug, but makes the binding of C5 slightly weaker and pH‐dependent [2]. After one induction dose, ravulizumab maintenance therapy is administered every 8 weeks making it far more convenient than eculizumab which is administered every 2 weeks. Ravulizumab was approved based on the positive results of the phase 3 CHAMPION MG trial [3].

A few months after ravulizumab became available, a substantial number of our patients who had switched from eculizumab to ravulizumab were reporting that ravulizumab was less effective than eculizumab in keeping their MG symptoms stable. We therefore started to employ therapeutic drug monitoring (TDM) under ravulizumab therapy to further understand these subjective reports.

For eculizumab, TDM has been established in other indications, namely atypical hemolytic uremic syndrome (aHUS) and paroxysmal nocturnal hemoglobinuria (PNH), by assessing classical complement pathway activity (CH50), alternative complement pathway activity (AH50) and drug level. Generally, a value below 10% for CH50 and AH50 is regarded as an acceptable and effective level of complement activity blockade [4, 5]. However, for monitoring ravulizumab therapy, measurement of serum‐free C5 has been proposed as a surrogate marker for complement blockade, with a cutoff of < 0.5 μg/mL indicating complete inhibition [3, 6, 7, 8]. Notably, the test is not commercially available, and its correlation with other inhibition markers remains unvalidated. Therefore, we chose to evaluate complement pathway activity under ravulizumab using the same approach as previously established for eculizumab.

This study aimed to explore CH50 and AH50 as biomarkers for ravulizumab therapy in gMG, and to investigate potential differences between ravulizumab and eculizumab, with the goal of optimizing treatment monitoring and guidance for C5‐inhibitor therapy.

## 2 Methods

### 2.1 Patients and Study Design

Patients with AChR‐ab positive gMG who received add‐on treatment with ravulizumab were recruited at the outpatient clinic of the integrated myasthenia center of the department of Neurology at the Charité Universitätsmedizin Berlin, Germany. Patients either switched from eculizumab to ravulizumab (“switchers”) or were C5‐inhibitor‐naïve before starting add‐on ravulizumab treatment. Blood samples and clinical scores were collected during routine clinical care and in most cases directly before the next ravulizumab or eculizumab infusion and therefore represent end‐of‐dosing interval status. Patient body weight was regularly monitored and documented; if changes were required, ravulizumab dosing was adjusted accordingly. At the end of each infusion, the tubing was flushed to ensure full administration of the prescribed dose. No measurements included in this analysis were taken from patients who had received IVIg or PLEX after initiation of C5‐inhibitor therapy. The study was conducted according to the Declaration of Helsinki and approved by the Ethics Committee of the Charité—Universitätsmedizin Berlin (EA1/281/10, EA1/214/18); written informed consent was given by all participants. This article follows the STrengthening the Reporting of Observational Studies in Epidemiology (STROBE) reporting guidelines.

### 2.2 Complement Pathway Activity Profiles and Drug Blood Levels

For TDM of C5‐inhibitors, the inhibition status of the complement system as well as C5‐inhibitor serum blood level was measured [9]. Complement inhibition status was assessed by determining CH50, AH50 and additionally, as surrogate markers for complement activation in vivo, levels of the complement activation products C3d (marker of complement activity upstream of C5) and sC5b‐9 (marker of terminal complement pathway activity). Blood samples (serum and EDTA plasma) were collected during routine clinical care, swiftly processed, and frozen at −80°C until shipment in batches on dry ice to the accredited Laboratory of Complement Diagnostics, Institute of Immunology, University Hospital Heidelberg, Germany.

CH50 was analyzed using a one‐point hemolytic test [10]. Serum was diluted 1:5 in gelatin veronal‐buffered saline containing 0.15 mM Ca^2+^ and 1 mM Mg^2+^ prior to the addition of antibody‐sensitized sheep erythrocytes (final serum dilution 1:15). The pH value of the buffer was 7.4–7.5 and the reference interval has been determined to be 74%–151%. For the analysis of AH50, the Wieslab Complement System Alternative Pathway immunoassay was employed (Svar Life Sciences, Malmö, Sweden). Serum samples were diluted 1:18 in the sample diluent provided in the kit. The pH value of the sample diluent was 7.35 and the reference interval has been determined to be 43%–107%.

Plasma concentrations of C3d, a stable cleavage fragment of complement component 3, were determined by employing double decker rocket immunoelectrophoresis [11]. Normal values are below 40 mU/L. The plasma concentration of sC5b9 was determined employing a commercial ELISA (Quidel Corporation, San Diego, USA). Normal values range between 58 and 239 ng/mL.

Serum levels of free eculizumab and ravulizumab were analyzed using in‐house ELISAs [9]. For eculizumab blood level, we used a cutoff of > 99 μg/mL [12] and for ravulizumab blood level a cutoff of > 175 μg/mL [13].

### 2.3 Clinical Scores and Patient‐Reported Subjective Ravulizumab Effect

During routine clinical care Myasthenia gravis Activities of Daily Living (MG‐ADL, 0–24 point score) [14], Myasthenia gravis Quality of Life, 15 items, revised version (MG‐QoL15r, 0–30 point score) [15, 16], and Quantitative Myasthenia Gravis score (QMG, 0–39 point score) [17] were assessed. We used all available scores for this analysis. Additionally, all patients were asked the following question: “How many weeks does the ravulizumab effect subjectively last?,” either after 6 months of treatment or at the time of decision to discontinue ravulizumab therapy.

### 2.4 Statistical Analysis

The statistical analyses were performed using R software (version 4.2.2) and R Studio software (version 2023.03.1 build 446). All analyses are realized using the full analysis set without any imputations. Descriptive statistics (mean, standard deviation, median, interquartile range, and absolute and relative frequencies) were applied to summarize patient characteristics and laboratory values where applicable. Effect sizes for group differences, i.e., Cohen's *d*, are calculated by estimates from linear mixed‐effects models for CH50, AH50, sC5b9 and C3d based on the log(*x* + 1)‐scale and for the level of ravulizumab and eculizumab concentration on the regular scale. Each model is adjusted for laboratory assessment type (or discontinuation of ravulizumab in the subgroup analysis), timepoint of blood assessment as week after the infusion, sex and age as fixed effects and random intercepts per available instance of measurement and per patient as crossed random effects since the timepoints of measurements differ between and within subjects. Group differences for outcomes with only one measurement, e.g., delta scores for patient‐related outcome measures (i.e., MG‐ADL, QMG, and QoL15r), were assessed as Cohen's *d* or odds ratio depending on the variable type. All effect sizes are reported with a 95% confidence interval. An effect size < 0.2 is regarded as negligible, 0.2–0.5 as small, 0.5–0.8 as moderate, and > 0.8 as large. A relevant difference between groups was assumed when the CI did not include zero. Relevant differences are highlighted in bold in the tables.

## 3 Results

**FIGURE 1:**
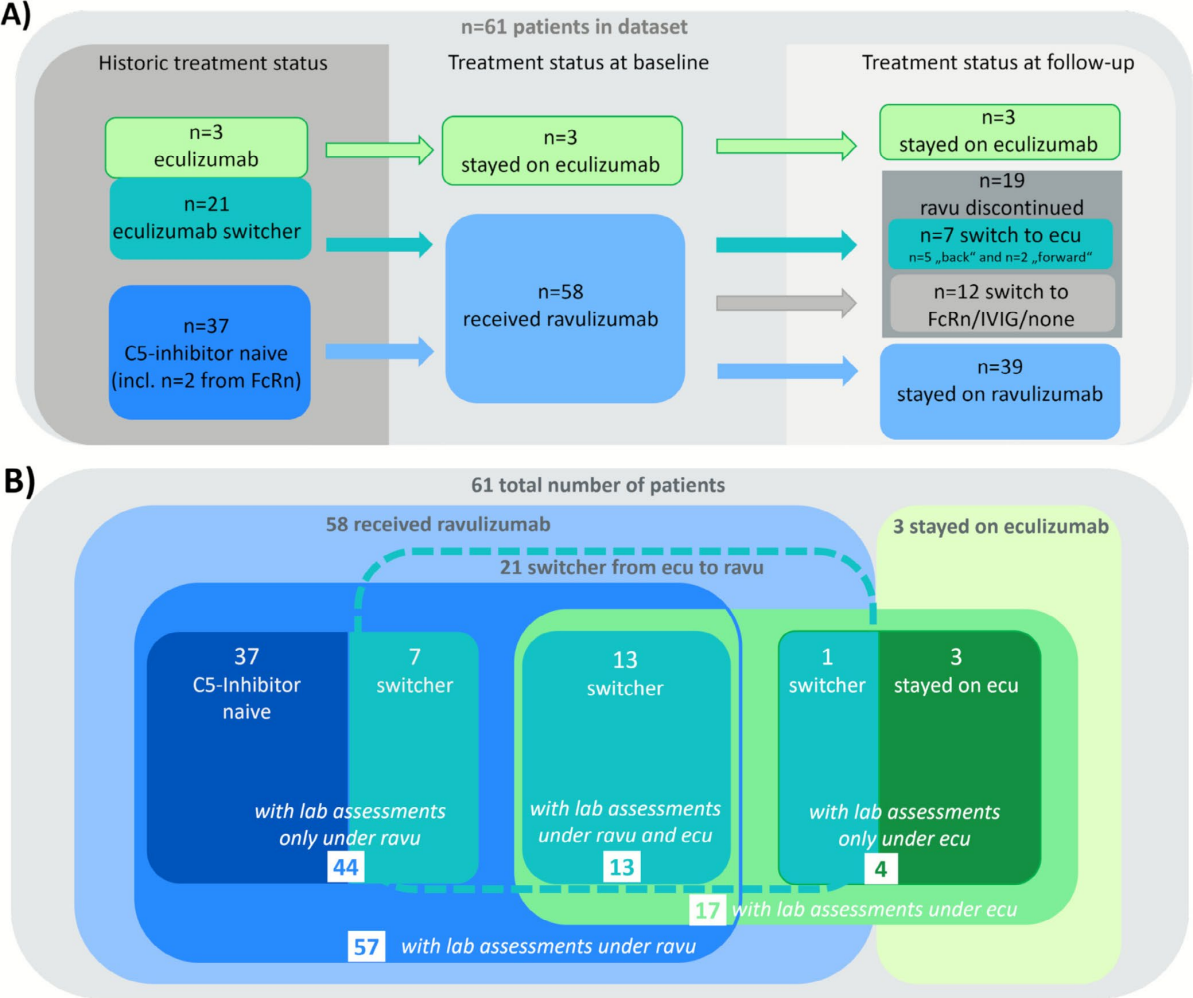
Therapies over time (A) and Venn diagram for patient subgroups (B). (A) Historic treatment status, treatment status at baseline and at follow‐up of the 61 patients in the cohort. A total of 58 were treated with ravulizumab. Of these, 37 were C5‐inhibitor naïve and 21 switched from eculizumab to ravulizumab. Three patients opted to remain on eculizumab throughout the analysis period. (B) Of the 58 patients who received ravulizumab 57 had lab assessments under ravulizumab therapy. A total of 17 patients had laboratory assessments under eculizumab therapy. This group consisted of the three patients who stayed on eculizumab, one patient who had switched from eculizumab to ravulizumab, but was only sampled after switching back to eculizumab and 13 patients who had switched from eculizumab to ravulizumab and were sampled under both eculizumab and ravulizumab therapy.

We prospectively included 61 patients who qualified for add‐on therapy with ravulizumab between November 2022 and April 2024. Of these, 24 patients who had previously been treated with eculizumab were offered to switch to ravulizumab, with 21 accepting and three opting to remain on eculizumab. Additionally, 37 C5‐inhibitor‐naïve patients initiated ravulizumab therapy. By the data cutoff in April 2024, three patients had remained on eculizumab, 39 patients continued ravulizumab and 19 patients had discontinued it, either switching (back) to eculizumab (*n* = 7) or transitioning to treatment with a FcRn inhibitor (*n* = 7), intravenous immunoglobulins (*n* = 2) or no add‐on therapy (*n* = 3) (Figure 1A and Figure S1).

### 3.1 Comparison Between Eculizumab and Ravulizumab

As a first step, we aimed to compare eculizumab and ravulizumab in their ability to suppress CH50 and AH50. We analyzed blood samples from 44 patients treated with ravulizumab alone and compared them to samples from 17 patients who had received eculizumab (Figure 1B). The exact timepoints and numbers of sampling per patient are shown in Figure S1. Both groups were comparable in age, sex distribution, body weight, duration of diagnosis, proportion of thymomas and history of thymectomy, although the eculizumab group had received a slightly higher number of previous immunotherapies (Table 1). Baseline characteristics for all subgroups shown in the Venn diagram in Figure 1B are listed in Table S1.

**TABLE 1:** Baseline characteristics of patients in the cohort.

	All patients with lab *N* = 61	Patients with only Ravu lab *N* = 44	Patients with Ecu lab *N* = 17
Female sex, *n* (%)	40 (65.6)	26 (59.1)	14 (82.4)
Age (yrs), median [IQR]	61.0 [43.0, 72.0]	61.0 [42.8, 72.5]	60.0 [46.0, 72.0]
Bodyweight (kg), median [IQR]	76.5 [63.3, 93.3]	77.0 [63.0, 96.5]	76.0 [63.5, 83.0]
Time since diagnosis (yrs), median [IQR]	6.0 [3.0, 10.0]	5.0 [3.0, 9.3]	9.0 [6.0, 10.0]
Thymectomy, *n* (%)	43 (70.5)	31 (70.5)	12 (70.6)
Thymoma, *n* (%)	9 (14.8)	5 (11.4)	4 (23.5)
Number of immunotherapies, median [IQR]	3.0 [3.0, 4.0]	3.0 [2.0, 3.0]	4.0 [3.0, 4.0]

Abbreviations: CI, confidence interval; Ecu, eculizumab; IQR, interquartile range; kg, kilogram; lab, laboratory assessment(s); Ravu, ravulizumab; yrs, years.

**FIGURE 2:**
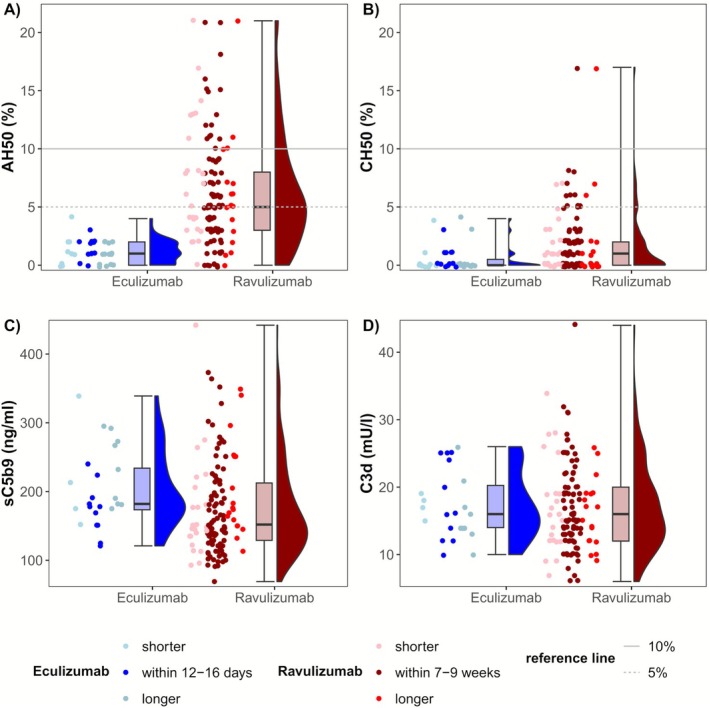
(A–D) Raincloud plots of AH50, CH50, C3d and sC5b9 under eculizumab and ravulizumab with differentiation into measurements within dosing intervals (for ravulizumab within 7–9 weeks and for eculizumab within 14 ± 2 days after last infusion) and outside of those intervals. For eculizumab dark blue dots represent measurements between 12 and 16 days after last infusion (34% of measurements). Lighter blue dots represent shorter intervals (< 12 days after last infusion) or longer intervals (> 16 days after last infusion). For ravulizumab dark red dots represent measurements between 7 and 9 weeks after last infusion (67% of measurements). Lighter red dots represent shorter intervals (< 7 weeks after last infusion) or longer intervals (> 9 weeks after last infusion).

Comparison of complement activity inhibition between the two groups revealed lower CH50 and AH50 levels under eculizumab than ravulizumab at the end of their respective maintenance therapy dosing intervals (2 and 8 weeks, respectively). The difference in the AH50 values between the groups was more pronounced and statistically significant (Cohen's *d*: 2.2; 95% CI [1.5–2.8]) than in the CH50 values (Cohen's *d*: 0.6; 95% CI [−0.1 to 1.3]). It is noteworthy that none of the patients receiving eculizumab had a CH50 or AH50 value greater than 4%, suggesting a profound suppression of complement pathway activity in all patients in the cohort. However, several patients on ravulizumab had AH50 values above 10%, with a maximum of 21%, indicating an only partial or too short‐lasting suppression of the alternative pathway in some patients (Table 2 and Figure 2; Table S2). A similar proportion of patients in both groups had C5‐inhibitor levels below the recommended blood level (Table 2). Ravulizumab but not eculizumab showed a tendency toward a blood‐level‐dependent suppression of AH50 and CH50 (Figure S2). Levels of C3d and sC5b9 were similar between groups (Table 2). The full distribution of samples and timepoints of collection relative to the prior infusion is shown in Figure S3. Additionally, we compared CH50 and AH50 in the 13 patients with measurements taken during both eculizumab and ravulizumab therapy. In all cases, AH50 values were lower under eculizumab, with a similar trend observed for CH50 values (Table S2 and Figure S4).

**TABLE 2:** CH50, AH50, C3d, sC5b9, and C5‐inhibitor levels under eculizumab and ravulizumab.

	All patients with lab *N* = 61	Patients with only Ravu lab *N* = 44	Patients with Ecu lab *N* = 17	Effect size	95% CI
CH50 (in %), median [IQR]	0.0 [0.0, 2.0]	1.0 [0.0, 2.0]	0.0 [0.0, 0.5]	0.6^a^	−0.1 to 1.3
AH50 (in %), median [IQR]	4.0 [1.8, 8.0]	5.0 [3.0, 9.0]	1.0 [0.0, 2.0]	**2.2** ^a^	**1.5 to 2.8**
sC5b9 (ng/mL), median [IQR]	169.0 [135.0, 218.0]	159.0 [124.0, 214.0]	182.0 [173.5, 234.0]	−0.5^a^	−1.3 to 0.3
C3d (mU/L), median [IQR]	16.0 [12.0, 20.0]	16.0 [12.0, 20.0]	16.0 [14.0, 20.3]	−0.1^a^	−1.0 to 0.8
Ravu‐level (μg/mL), median [IQR]	—	295.5 [201.5, 411.8]	—	—	—
Ravu‐level < 175 μg/mL	—	10 (22.7%)	—	—	—
Ecu‐level (μg/mL), median [IQR]	—	—	300.0 [168.0, 378.0]	—	—
Ecu‐level < 99 μg/mL	—	—	3 (17.6%)	—	—

*Note*: Normal values for sC5b9: 58–239 ng/mL; normal values for C3d < 40 mU/L.

Abbreviations: AH50, alternative complement pathway activity; CH50, classical complement pathway activity; CI, confidence interval; Ecu, eculizumab; IQR, interquartile range; lab, laboratory assessment(s); Ravu, ravulizumab.

^a^ Cohen's *d*; Effect size is highlighted in bold if the CI does not include zero and is therefore interpreted as a relevant group difference.

### 3.2 Complement Pathway Activity Levels Were Higher in Ravulizumab Non‐Responders

Of the 58 patients who had started ravulizumab therapy, 19 (32.7%) discontinued therapy due to a lack of or waning effect within 6 weeks or less after the last infusion (Figure 1A). The decision to discontinue ravulizumab was based on the shared decision‐making of the treating physicians and the patients, regardless of the individual CH50/AH50 levels and was taken after a minimum of 2 and a maximum of 7 total infusions. One patient was sampled only after switching back to eculizumab and not sampled while on ravulizumab therapy and was therefore excluded from this analysis. The baseline characteristics of the 39 patients who continued and the 18 patients who discontinued ravulizumab, for whom samples were collected while on ravulizumab, were comparable (Table 3). Two‐thirds of the patients who were switched from previous eculizumab therapy to ravulizumab were in the group that continued to use ravulizumab. However, the proportion within the two groups (continued versus discontinued) was the same at about one‐third in each group (Table 3).

**TABLE 3:** Baseline characteristics of patients who continued versus discontinued ravulizumab therapy.

	Ravulizumab all *N* = 57	Ravulizumab continued *N* = 39	Ravulizumab discontinued *N* = 18
Female sex, *n* (%)	37 (64.9%)	24 (61.5%)	13 (72.2%)
Age (yrs), median [IQR]	61.0 [43.0, 72.0]	61.0 [45.0, 74.5]	57.5 [43.0, 69.5]
Bodyweight (kg), median [IQR]	76.0 [63.5, 94.5]	73.5 [59.8, 90.8]	80.0 [74.0, 106.0]
Yrs since diagnosis, median [IQR]	6.0 [3.0, 10.0]	8.0 [3.0, 9.0]	5.0 [3.3, 10.8]
Thymectomy, *n* (%)	39 (68.4%)	25 (64.1%)	14 (77.8%)
Thymoma, *n* (%)	8 (14.0%)	5 (12.8%)	3 (16.7%)
Number of immunotherapies, median [IQR]	3.0 [3.0, 3.0]	3.0 [2.5, 4.0]	3.0 [3.0, 3.0]
Switcher from eculizumab, *n* (%)	20 (27.0%)	14 (35.9%)	6 (33.3%)

Abbreviations: CI, confidence interval; IQR, interquartile range; kg, kilogram; Ravu, ravulizumab; yrs, years.

**FIGURE 3:**
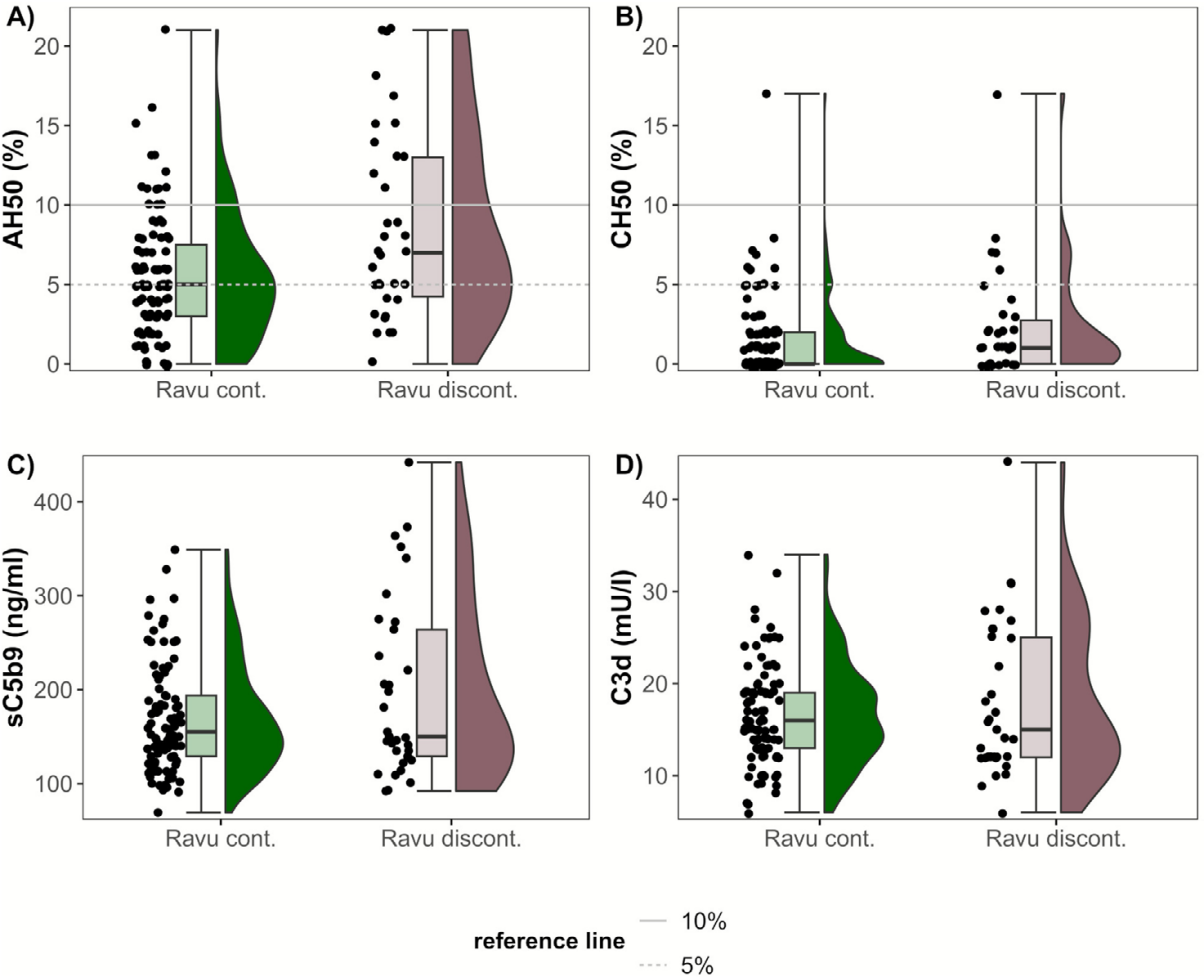
(A–D) Comparison of AH50, CH50, sC5b9, and C3d of patients who continued versus discontinued ravulizumab therapy. Raincloud plots of AH50, CH50, sC5b9, and C3d of the group that continued ravulizumab (Ravu cont.; green color) and the group that discontinued ravulizumab (Ravu discont.; red color).

The group that continued ravulizumab showed lower median values for AH50 with a large effect size (Cohen's *d*: −0.9; 95% CI [−1.3 to −0.4]) compared to the group that discontinued therapy. For CH50 there was a statistically non‐robust trend toward lower values in the group that continued ravulizumab. Levels of C3d and sC5b9 were similar between groups (Table 4 and Figure 3). One‐fifth of patients had ravulizumab levels below the recommended value of 175 μg/mL indicating heterogeneous and individual pharmacokinetics. However, this was equally distributed between those who continued ravulizumab therapy and those who discontinued it (Table 4 and Figure S5). Of all samples analyzed, 67% were collected within 7–9 weeks after the last ravulizumab infusion and 66% of patients were sampled at 2–3 different timepoints. The full distribution of samples and timepoints of collection relative to the prior infusion is shown in Figure S5.

**TABLE 4:** Levels of complement pathway activity, complement factors and C5 inhibitors, change in clinical scores, subjective duration of ravulizumab effect and time to follow‐up of patients who continued versus discontinued ravulizumab therapy.

	Ravulizumab all *N* = 57	Ravulizumab continued *N* = 39	Ravulizumab discontinued *N* = 18	Effect size	95% CI
Laboratory results					
CH50 (in %), median [IQR]	1.0 [0.0, 2.0]	0.0 [0.0, 2.0]	1.0 [0.0, 2.8]	−0.4^b^	−0.9 to 0.1
AH50 (in %), median [IQR]	5.0 [3.0, 8.0]	5.0 [3.0, 7.5]	7.0 [4.3, 13.0]	**−0.9** ^b^	**−1.3 to −0.4**
sC5b9 (ng/mL), median [IQR]	152.0 [129.0, 212.5]	155.0 [129.0, 193.8]	150.0 [129.0, 264.0]	−0.5^b^	−1.0 to 0.1
C3d (mU/L), median [IQR]	16.0 [12.0, 20.0]	16.0 [13.0, 19.0]	15.0 [12.0, 25.0]	−0.2^b^	−0.8 to 0.3
Ravu‐level (μg/mL), median [IQR]	294.0 [239.0, 410.0]	317.0 [245.0, 408.0]	267.0 [233.3, 431.3]	0.4^b^	−0.2 to 0.4
Ravu‐level < 175 μg/mL, *n* (%)	11 (19.3%)	7 (17.9%)	4 (22.2%)	0.8^a^	0.2–3.0
Clinical scores					
Δ MG‐ADL, median [IQR]	0.0 [−1.0, 1.0]	0.0 [−2.0, 1.0]	0.0 [−1.0, 1.5]	−0.4^b^	−1.0 to 0.2
Missing	6	3	3		
Δ QMG, median [IQR]	1.0 [−2.0, 3.0]	2.0 [−2.0, 3.0]	−0.5 [−1.5, 2.0]	0.2^b^	−0.5 to 0.8
Missing	10	4	6		
Δ QoL15r, median [IQR]	0.0 [−2.0, 2.0]	−1.0 [−3.8, 1.0]	1.5 [0.8, 4.0]	**−0.7** ^b^	**−1.3 to 0.0**
Missing	11	5	6		
Patient‐reported subjective Ravu effect^c^ (weeks), median [IQR]	6.0 [3.0, 7.1]	7.0 [6.0, 8.0]	0.0 [0.0, 4.0]	**2.3** ^b^	**1.5–3.0**
Missing	5	4	1		
Weeks BL to FU, median [IQR]	26.6 [24.7, 27.4]	27.0 [26.0, 27.8]	19.0 [12.9, 26.2]	**0.8** ^b^	**0.2–1.4**

*Note*: Normal values for sC5b9: 58–239 ng/mL; normal values for C3d < 40 mU/L.

Abbreviations: AH50: alternative complement pathway activity; BL: baseline; CH50: classical complement pathway activity; CI: Confidence interval; Δ = delta or change in score between follow‐up after 6 months and baseline; FU: follow‐up; IQR: interquartile range; kg: kilogram; Ravu: ravulizumab; yrs: years.

^a^ Odds ratio.

^b^ Cohen's *d*; Effect size is highlighted in bold if the CI does not include zero and is therefore interpreted as a relevant group difference.

^c^ Answer to the question: “How many weeks does the ravulizumab effect subjectively last?”

In addition to the laboratory data, we collected clinical data at baseline before the start of ravulizumab therapy and after 6 months of ravulizumab therapy or at the time of discontinuation if that was earlier than 6 months. Consequently, the median time to follow‐up was shorter in the group that discontinued ravulizumab therapy, with a median of 19 weeks (Table 4). At baseline, the median MG‐ADL score was 10 points and the median QMG score was 16 points (Table S3). At follow‐up, the median changes in the MG‐ADL and QMG scores were between 0 and 2 points in both groups. The median change in the MG‐QoL15r score showed a slight improvement in the group that continued ravulizumab treatment compared to a slight deterioration in the group that discontinued treatment (Cohen's *d*: −0.7; 95% CI [−1.3 to 0.0]). The most pronounced difference between the two groups was the patient‐reported subjective duration of ravulizumab effect (answer to the question: “How many weeks does the ravulizumab effect subjectively last?”), with a median duration of 7 weeks in the group that continued treatment versus 0 weeks in the group that discontinued (Cohen's *d*: 2.3; 95% CI [1.5–3.0]) (Table 4).

**FIGURE 4:**
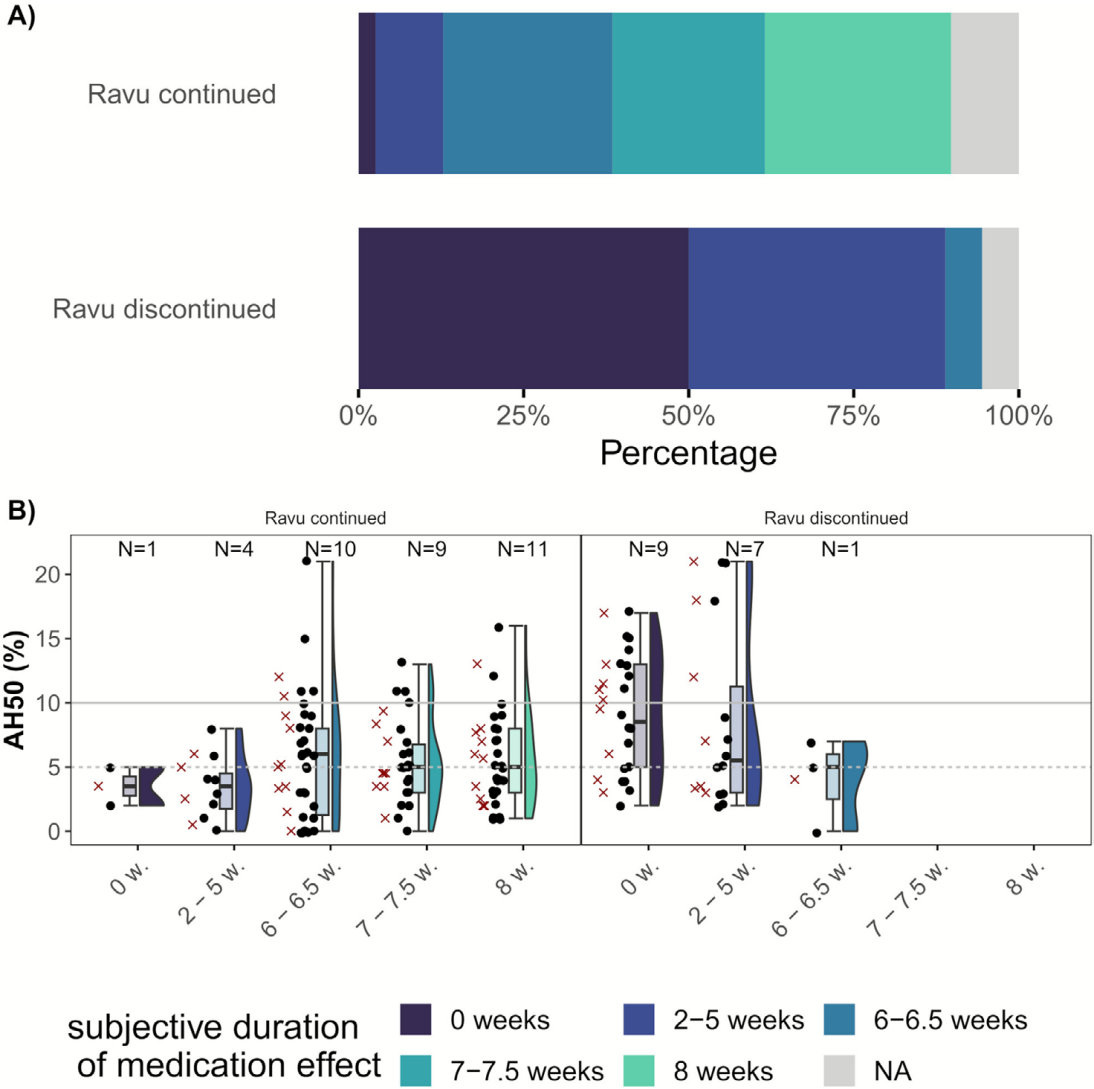
(A) Stacked bar charts of subjective duration of ravulizumab effect (in weeks) and (B) Raincloud plots for AH50 at end‐of‐dose interval for duration of patient‐reported subjective ravulizumab effect for patients who continued versus discontinued ravulizumab therapy. Patients answered the question: “How many weeks does the ravulizumab effect subjectively last?” The dots in the raincloud plots represent single lab assessments. The x in the raincloud plots represent the mean AH50 per patient from several measurements as 66% of patients were sampled two or three times (exact distribution of sampling presented in Figure S1). Lab assessments were mostly done at the end of the dosing interval, which was the standard 8‐week interval.

Of all patients examined, only 11 (19%) patients reported a subjective duration of ravulizumab effect of 8 weeks (Figure 4, Table S4). All 11 patients belong to the group that continued ravulizumab therapy and account for 28% of this subgroup. In the group that discontinued therapy, half of the patients reported a subjective duration of ravulizumab effect between 2 and 6 weeks. The other half reported that ravulizumab had not worked at all, meaning that their MG symptoms did not improve or worsened after the start of ravulizumab therapy. These ten patients could be defined as nonresponders to ravulizumab. In a visualization of the duration of patient‐reported effect of ravulizumab with corresponding AH50 values (Figure 4), the highest median value of AH50 is found in the subgroup of nonresponders.

## 4 Discussion

Our study suggests that ravulizumab suppresses complement activity to a lesser extent than eculizumab and that a subgroup of patients on ravulizumab treatment has a higher level of residual activity of the alternative pathway as measured by AH50 values. In particular, higher AH50 values were correlated with a faster decline in the therapeutic effect of ravulizumab as perceived by patients.

In contrast to other complement‐mediated diseases such as PNH, it has been hypothesized that the alternative pathway does not play an important role in MG. However, our results suggest that certain functional aspects of the alternative pathway may play an important role, at least in a subgroup of patients. First, the alternative pathway is constantly and autonomously activated at a low‐level [18], and there might be individual differences in this low‐level activation. Second, the alternative pathway entails an amplificatory loop proximal to the activation of C5 that utilizes C3b, factors D, and B, thereby amplifying C3b production [19]. The binding of C3b to C5 induces a steric conformational change that is necessary for the cleavage of C5 and thus for activation, which underlines the importance of C3b for the activation of the terminal complement pathway. Overall, the alternative pathway contributes substantially to the overall complement activation—including the part mediated by the classical pathway [20]. Moreover, laboratory data indicates that excess amounts of clustered C3b molecules bound to surfaces might compete to some extent with C5 inhibitors and that this may result in a residual level of C5 activation [18]. Therefore, the alternative pathway may play a more important role in the activation of the terminal complement cascade than is generally assumed [20]. Our data suggests that this may also play a role in MG. This assumption is supported by data showing increased levels of Ba and Bb in AChR‐ab positive patients with gMG [21]. Taking into account studies suggesting that even very low concentrations of unbound or free C5 produce up to 20% detectable complement pathway activity [18, 22], even a small rerelease of C5 from ravulizumab could be sufficient to explain the relatively high AH50 levels in some of our patients. In our opinion, this warrants further research into the role of the alternative complement pathway in MG.

Overall, there are several challenges in TDM for C5‐inhibitors. First, available C5 assays cannot distinguish between free and inhibitor‐bound C5, resulting in falsely elevated C5 levels under therapy with C5‐inhibitors [12, 23]. Moreover, the cleavage product C5a has an extremely short half‐life (< 1 h), requiring immediate freezing for reliable measurement, which limits its practical use [12]. For eculizumab, TDM has been established as an assessment of CH50, AH50, and drug level [24, 25, 26]. Regarding ravulizumab however, there is a further concern: due to the pH‐dependent binding of ravulizumab to C5, it has been hypothesized that some of the commercially available CH50 assays that are performed at a low pH in vitro could lead to inaccurate results under ravulizumab [12, 27]. This might be one of the reasons, why instead of monitoring ravulizumab therapy with CH50, the measurement of free C5 has been introduced [8]. However, the Gyros‐based fluorescence assay (Smithers Avanza, Gaithersburg, USA [23]) used in the phase 3 trials to measure free C5 is not available at routine complement laboratories due to its very high cost. A free C5 value of < 0.5 μg/mL has been defined as a cutoff to indicate complete terminal complement inhibition [3, 6, 7]. However, the evidence that this correlates with other measurements of terminal complement inhibition is lacking. In contrast, recent data show that a low‐level of free C5 corresponds to a CH50 value of up to 25% [27, 28, 29], which is considered only a partial blockade of complement activity.

In this study, we employed a standard sheep red blood cell (SRBC)‐based CH50 assay as well as an ELISA‐based AH50 assay [12] for TDM of eculizumab and ravulizumab. Both these assays work at a physiological pH level ruling out a potential increased release of C5 by ravulizumab vs. eculizumab due to low pH. Our results show that eculizumab treatment suppressed CH50 and AH50 reliably in all patients to ≤ 4%, while AH50 activities of > 10% were observed in a significant number of patients under ravulizumab treatment. This difference in inhibitory effects of both drugs on AH50 and—to a lesser extent on CH50—under the same assay conditions and at similar concentrations (and when measured in the same patient) may be explained by the fact that even at pH 7.4 the affinity of ravulizumab to C5 is still lower than that of eculizumab [2].

Specifically in patients with gMG, target values for CH50 and AH50 are not clearly defined for TDM under C5‐inhibitor therapy. Under eculizumab, a value below 10% for CH50 and also AH50 is considered an acceptable and effective degree of complement activity blockade [4, 5]. As discussed above, for ravulizumab, apart from our own data, there is only scarce data on CH50 and AH50 levels. However, for zilucoplan, the same cutoffs for the SRBC‐based CH50 assay have been used as for eculizumab [30, 31]. Data from the phase 2 trial of zilucoplan suggest that levels of suppression lower than 10% may further improve clinical outcomes in MG. For zilucoplan, the improvement in clinical outcomes was slower and less pronounced for the lower dose (0.1 mg/kg) than for the higher dose (0.3 mg/kg). Both doses suppressed CH50 to levels below 15%, but the higher dose achieved a nearly complete suppression of complement activity with CH50 values near 0% after 2 weeks of therapy [30]. Additionally, laboratory data using C5‐deficient serum suggest that as little as 1%–2% of unblocked C5 in serum can lead to measurable complement activity [22] and that concentrations of free C5 of 0.1 μg/mL already lead to AH50 values of 20% residual activity [18]. Taken together, these studies suggest that the target for complement activity suppression should be well below 10% of residual activity and potentially as close to 0% as possible.

This notion is supported by our data obtained from the subgroup of 13 patients with measurements under both eculizumab and ravulizumab: there were three patients who had acceptable AH50 and CH50 values below 10% on ravulizumab but reported worsening of MG symptoms or rapid wearing off. After switching back to eculizumab, they had AH50 and CH50 values below 5% and reported clinical stabilization or improvement. These three patients might represent a subgroup of MG patients for whom blocking complement activity at levels near 0% correlates with a better outcome. This hypothesis is further corroborated by data from a phase‐3b trial in which patients were switched from either eculizumab or ravulizumab to zilucoplan. At baseline, patients previously treated with eculizumab had mean CH50 levels of 3%, while patients previously treated with ravulizumab had mean CH50 levels of 13%. In the latter subgroup, CH50 levels dropped to 1%–2% under zilucoplan, correlating with clinical improvement in MG‐ADL and QMG [31].

From a clinical perspective, it is surprising that 81% of patients reported a wearing off of subjective ravulizumab effect (or a recurrence of symptoms) several weeks before the next infusion despite a regular 8‐week interval between maintenance infusions. The available pharmacokinetic data for eculizumab and ravulizumab may provide a partial explanation. For eculizumab, a mean elimination half‐life of 18.2 ± 6.3 days (mean ± SD) was reported in patients with gMG, corresponding to a mean of 2.6 weeks. The reported minimum was 5.6 days and the maximum was 39.6 days [29]. The 2‐week interval for the eculizumab therapy is therefore well below the mean elimination half‐life. The wide range between less than a week and more than a month is consistent with studies in pediatric and adult patients with aHUS and PNH that have unveiled large interindividual differences in pharmacokinetics and enabled individualized (prolonged) dosing regimens [22, 24, 32, 33]. In contrast, the reported mean elimination half‐life of ravulizumab in patients with gMG was 56.6 ± 8.3 days (mean ± SD), which corresponds to a mean of 8.1 weeks. The minimum and maximum elimination half‐lives were not reported [34]. In patients with PNH and aHUS, the mean elimination half‐life of ravulizumab was 49.6 ± 9.1 and 51.8 ± 16.2 (mean ± SD), respectively, corresponding to a mean of 7 weeks [23, 35]. The shorter mean elimination half‐life in patients with PNH and aHUS could be explained by the proportion of pediatric patients in these cohorts, since children generally tend to metabolize drugs faster. However, apart from this, the data suggest that the 8‐week maintenance interval may be too long for a relevant proportion of patients. The results from our exploratory study support this conclusion. A similar effect was reported in Japan, where within a group of 14 switchers ravulizumab was preferred over eculizumab overall and for convenience but not regarding effectiveness [36].

However, the data from this study should not be used to make assumptions on the clinical effectiveness of ravulizumab therapy in general, since it was an exploratory design and focused on biomarkers. Moreover, our study has important limitations. These include the heterogeneity in the timing of the clinical scoring. We generally did not assess the scores at a fixed time point, nor at the time of best effect but after the symptoms recurred. Thus, the clinical effect of ravulizumab is likely underestimated. The decision to continue or discontinue ravulizumab therapy was based on shared decision‐making between the physician and patient and was based on the patient's subjective assessment of the effects after the infusions. It was not based on prespecified changes in MG‐ADL or QMG scores. However, it should also be noted when interpreting the data that the complement activity data were not included in the decision‐making process. The fact that patients continued ravulizumab therapy at the time of data collection despite clinical worsening or symptom recurrence from 0 to 5 weeks after the last infusion indicates that the decision to discontinue therapy was not taken lightly and usually involved review of several treatment cycles. Moreover, assessment of the clinical scores was partially incomplete and in several cases patients who had discontinued ravulizumab were assessed at the last visit when they were stable and not during the exacerbation of MG symptoms that led to discontinuation of ravulizumab therapy. Finally, the individual changes in clinical scores were very heterogeneous, with almost as many patients experiencing a worsening as those experiencing an improvement, leading to a leveling of group effects. Further limitations include the exploratory nature of the analysis in a small number of patients and the fact that time points and the number of blood samples taken per patient were heterogeneous. To correct for the latter, we made statistical adjustments for group comparisons, as described in the methods section. Moreover, we acknowledge that undetected antidrug antibodies could potentially contribute to a reduced effect of ravulizumab in individual patients. However, in a subanalysis of data from the CHAMPION MG trial, antidrug antibodies were detected only prior to treatment and not during therapy in 86 patients receiving ravulizumab. No neutralizing antibodies were observed, indicating a low immunogenic potential of ravulizumab [34]. Further, this study did not assess the impact of IVIg or PLEX on complement activity or C5‐inhibitor levels, although this may represent an important question in clinical routine.

Overall, our cohort was similar to the study participants of the CHAMPION MG trial [3]. However, our cohort included a slightly larger proportion of females (65% in our cohort vs. 51% in CHAMPION MG) and the disease duration was shorter in our cohort (6 years in our cohort versus 10 years in CHAMPION MG). Baseline MG‐ADL and QMG scores were very similar, despite one‐third of our patients having been pretreated with eculizumab. The minimally clinically important differences (MCIDs) for MG‐ADL and QMG were achieved by 64% and 45% of patients, respectively, in the CHAMPION MG trial [3]. A post hoc analysis of the CHAMPION MG trial data indicated that some patients exhibited a delayed response, resulting in higher overall response rates [37]. Regardless, these data are consistent with 33% of patients in our cohort who discontinued ravulizumab therapy due to lack of effectiveness or wearing off several weeks prior to the next infusion.

## 5 Conclusion

This study provides three important findings that need to be validated in large, multicenter biomarker studies. First, in this cohort of MG patients ravulizumab revealed less complete complement activity suppression than eculizumab, which underscores the need for further comparisons, including studies with zilucoplan. Second, our data suggest a more important role of the alternative complement pathway in MG than generally thought, at least for a subgroup of patients. This warrants further investigations into the role of the alternative complement pathway in MG and maybe even therapeutic investigations, such as exploring a combination of ravulizumab with factor D or factor B inhibitors. Third, optimizing the dosing interval of ravulizumab is critical to maximizing its therapeutic effect. While ravulizumab is effective in most patients, some patients require shorter dosing intervals, with AH50 serving as a potential predictive biomarker. Conversely, extending dosing intervals could be an option for selected patients receiving eculizumab.

## Author Contributions

L.G. wrote the manuscript. L.G. and A.M. developed the study design. L.G. and F.S. conducted the study. L.G., S.L., and A.M. interpreted the data. Statistical analyses were performed by M.M. The manuscript was critically reviewed and edited by all authors.

## Conflicts of Interest

L.G. received Speaker's honoraria and/or travel and congress fees from Alnylam, Alexion, Roche and UCB and is a shareholder of RareLink digital health GmbH. F.S. received travel/accommodation/meeting expenses from Alexion Pharmaceuticals and Argenx and received speaking honoraria and honoraria for attendance at advisory boards from Alexion Pharmaceuticals, Argenx and UCB pharma. She receives financial research support (paid to her institution) from Alexion Pharmaceuticals and Argenx. She serves as a member of the medical advisory board of the German Myasthenia Gravis Society e.V. P.D. has received honoraria for attendance at advisory boards for UCB and speaker's honoraria from Alexion and Argenx. C.D. received speaker's honoraria from Alexion and UCB; and travel/accommodation/meeting expenses and honoraria for attendance at advisory boards from Argenx. M.H. has received speaker's honoraria from Argenx and speaker's honoraria and honoraria for attendance at advisory boards from Alexion. M.S. has received speaker's honoraria and honoraria for attendance at advisory boards from Argenx and Alexion and is a shareholder of RareLink digital health GmbH. P.M. received travel/accommodation/meeting expenses from UCB pharma. J.D.L. has received honoraria for acting as a member of Scientific Advisory Boards for Abbvie, Astra Zeneca Rare Disease/Alexion, Argenx, Johnson & Johnson, Moderna, Roche Pharma AG, Sanofi, Takeda, and UCB as well as speaker honoraria and travel support from Astra Zeneca Rare Disease/Alexion, Argenx, Biogen, CSL Behring, Roche Pharma AG, Merck, Moderna, Novartis, Octapharma, and Sanofi. His research is funded by the European Union, Deutsche Forschungsgesellschaft (DFG), Alexion, Argenx, Moderna, and Roche Pharma AG, and is a member of the medical advisory board of the German MG Society. J.S.‐B. has received honoraria for attendance at advisory boards from Novartis. S.L. received travel/accommodation/meeting expenses from Alexion Astra Zeneca Rare Disease, Argenx, Johnson & Johnson, and UCB; she received speaking honoraria and honoraria for attendance at advisory boards from Alexion Astra Zeneca Rare Disease, Argenx, Biogen, Hormosan, Huma, Johnson & Johnson, Merck, Roche, StreamedUp and UCB. She received financial research support (paid to her institution) from Ad Scientiam, Alexion Pharmaceuticals, Argenx, Hormosan and UCB. SL is a shareholder of RareLink digital health GmbH and mamahealth GmbH. S.H. has received speaker honoraria from Alexion, Argenx BV, Grifols, Johnson & Johnson, Merck, Roche, UCB; honoraria for attendance at advisory boards from Alexion, Argenx BV, Johnson & Johnson, Novartis, and Roche, research grants from Argenx BV and Johnson & Johnson and serves as a member of the medical advisory board of the German Myasthenia Gravis Society. A.M. received speaker's honoraria, served as an advisory board/DSMB member and consultant, and has received research grants (paid to his institution) from Alexion AstraZeneca Rare Disease, Argenx, Axunio, Desitin, Genpharm, Grifols, Hormosan, Immunovant, Janssen, Merck, Novartis, Octapharma, Regeneron, Sanofi and UCB. He served as chairman of the medical advisory board of the German Myasthenia Gravis Society. M.M., A.S., G.W. report no conflicts of interest.

## Supporting information


**Appendix S1:** acn370251‐sup‐0001‐AppendixS1.docx.

**Figure S1:** acn370251‐sup‐0002‐FigureS1.tif.

**Figure S2:** acn370251‐sup‐0003‐FigureS2.tif.

**Figure S3:** acn370251‐sup‐0004‐FigureS3.tif.

**Figure S4:** acn370251‐sup‐0005‐FigureS4.tif.

**Figure S5:** acn370251‐sup‐0006‐FigureS5.tif.

## Data Availability

The data that support the findings of this study are available from the corresponding author upon reasonable request.
