# Supplementary material for: Inhibition of Classical and Alternative Complement Pathway by Ravulizumab and Eculizumab
Source: Ann Clin Transl Neurol. 2025 Nov 19;13(4):688–99. doi: 10.1002/acn3.70251 (PMC13071113; doi:10.1002/acn3.70251)
Supplement: Supplementary file 1 — Appendix S1: acn370251‐sup‐0001‐AppendixS1.docx. [file ACN3-13-688-s004.docx]

**Supplementary data**

to Manuscript entitled:

**Inhibition of classical and alternative complement pathway by ravulizumab and eculizumab**

Authors:

Lea Gerischer^1,2^, MD, Frauke Stascheit^1,2^, MD, Maximilian Mönch^3^, Paolo Doksani^1,2^, MD, Carla Dusemund^1,2^, Meret Herdick^1,2^, MD, Philipp Mergenthaler^1,4,8^, MD, Maike Stein^1,2^, MD, Amani Suboh^1,2^, MD, Jutta Schröder-Braunstein^6^, MD, Guido Wabnitz^6^, MD, Jan D. Lünemann^7^, MD, Sophie Lehnerer^1,2,5^, MD, Sarah Hoffmann^1,2^, MD, Andreas Meisel^1,2,4^*,MD,

^1^ Department of Neurology with Experimental Neurology and integrated Myasthenia Center, Charité – Universitätsmedizin Berlin, corporate member of Freie Universität Berlin and Humboldt-Universität zu Berlin, Berlin, Germany

^2^ Neuroscience Clinical Research Center, Charité – Universitätsmedizin Berlin, corporate member of Freie Universität Berlin and Humboldt-Universität zu Berlin, Berlin, Germany

^3^ Institute of Biometry and Clinical Epidemiology, Charité – Universitätsmedizin Berlin, corporate member of Freie Universität Berlin and Humboldt-Universität zu Berlin, Berlin, Germany

^4^ Center for Stroke Research Berlin, Charité – Universitätsmedizin Berlin, Berlin, Germany

^5^ Berlin Institute of Health at Charité – Universitätsmedizin Berlin, Digital Health Center, Berlin, Germany

^6^ Institute of Immunology, Laboratory of Complement Diagnostics, University Hospital Heidelberg, Heidelberg, Germany

^7^ Department of Neurology, University Hospital Münster, Münster, Germany

^8^ Radcliffe Department of Medicine, University of Oxford, Oxford, UK

**Supplementary Table 1.**

| **Supplementary Table 1. Baseline characteristics of all subgroups in the cohort** | | | | | | |
| --- | --- | --- | --- | --- | --- | --- |
|  | **all patients**  **with lab**  N=61 | **all patients with ravu lab**  N=57 | **patients with only ravu lab**   N=44 | **patients with  ravu and ecu lab**  N=13 | **patients with only ecu lab**   N=4 | **all patients with ecu lab**   N=17 |
| **Female sex**, n (%) | 40 (65.6%) | 37 (64.9%) | 26 (59.1%) | 11 (84.6%) | 3 (75.0%) | 14 (82.4%) |
| **Age (yrs),**  median [IQR] | 61.0  [43.0, 72.0] | 61.0  [43.0, 72.0] | 61.0  [42.8, 72.5] | 60.0  [46.0, 72.0] | 55.5  [44.3, 68.0] | 60.0  [46.0, 72.0] |
| (min,max) | (22.0,88.0) | (22.0,88.0) | (22.0,88.0) | (24.0,85.0) | (36.0,80.0) | (24.0,85.0) |
| **Bodyweight (kg),**  median [IQR] | 76.5  [63.3, 93.3] | 76.0  [63.5, 94.5] | 77.0  [63.0, 96.5] | 76.0  [63.8, 82.5] | 77.0  [68.5, 83.5] | 76.0  [63.5, 83.0] |
| (Min,Max) | (47.0,129.0) | (47.0,129.0) | (47.0,129.0) | (52.0,110.0) | (60.0,90.0) | (52.0,110.0) |
| **Yrs since diagnosis,**  median [IQR] | 6.0  [3.0, 10.0] | 6.0  [3.0, 10.0] | 5.0  [3.0, 9.3] | 9.0  [7.0, 10.0] | 8.0  [5.8, 14.0] | 9.0  [6.0, 10.0] |
| (Min,Max) | (1.0,31.0) | (1.0,31.0) | (1.0,31.0) | (3.0,17.0) | (5.0,26.0) | (3.0,26.0) |
| **Thymectomy,** n (%) | 43 (70.5%) | 39 (68.4%) | 31 (70.5%) | 8 (61.5%) | 4 (100.0%) | 12 (70.6%) |
| **Thymoma,** n (%) | 9 (14.8%) | 8 (14.0%) | 5 (11.4%) | 3 (23.1%) | 1 (25.0%) | 4 (23.5%) |
| **Number of immunotherapies,**  median [IQR] | 3.0  [3.0, 4.0] | 3.0  [3.0, 3.0] | 3.0  [2.0, 3.0] | 4.0  [3.0, 4.0] | 3.5  [2.8, 4.0] | 4.0  [3.0, 4.0] |
| (Min,Max) | (2.0,5.0) | (2.0,5.0) | (2.0,5.0) | (2.0,5.0) | (2.0,4.0) | (2.0,5.0) |
| abbreviations: CI: Confidence interval; Ecu: eculizumab; IQR: inter-quartile range; kg: kilogram; lab: laboratory assessment(s); Max: maximum; Min: minimum; Ravu: ravulizumab; yrs: years. | | | | | | |

**Supplementary Table 2.**

| **Supplementary Table 2.**  **CH50, AH50, C3d, sC5b9 and C5-inhibitor levels under eculizumab and ravulizumab for all subgroups** | | | | | | |
| --- | --- | --- | --- | --- | --- | --- |
|  | **All Patients with Ravu lab**  N=57 | **Patients with only Ravu lab**  N=44 | **Patients with  Ravu and Ecu lab**  N=13 | | **Patients with only Ecu lab**    N=4 | **All Patients with Ecu lab**    N=17 |
|  |  |  | **Patients with Ravu Lab** | **Patients with Ecu Lab** |  |  |
| **CH50** (in %), median [IQR] | 1.0  [0.0, 2.0] | 1.0  [0.0, 2.0] | 1.0  [0.0, 2.0] | 0.0  [0.0, 1.0] | 0.0  [0.0, 0.0] | 0.0  [0.0, 0.5] |
| (Min,Max) | (0.0,17.0) | (0.0,17.0) | (0.0,7.0) | (0.0,4.0) | (0.0,3.0) | (0.0,4.0) |
| **AH50** (in %), median [IQR] | 5.0  [3.0, 8.0] | 5.0  [3.0, 9.0] | 4.5  [3.0, 6.0] | 1.0  [0.0, 2.0] | 1.0  [0.3, 1.8] | 1.0  [0.0, 2.0] |
| (Min,Max) | (0.0,21.0) | (0.0,21.0) | (0.0,21.0) | (0.0,4.0) | (0.0,2.0) | (0.0,4.0) |
| **sc5b9** (ng/ml), median [IQR] | 152.0  [129.0, 212.5] | 159.0  [124.0, 214.0] | 151.0  [135.3, 196.0] | 178.0  [160.5, 201.5] | 267.0  [182.0, 292.0] | 182.0  [173.5, 234.0] |
| (Min,Max) | (69.0,442.0) | (91.0,442.0) | (69.0,328.0) | (121.0,240.0) | (151.0,339.0) | (121.0,339.0) |
| **C3d** (mU/l), median [IQR] | 16.0  [12.0, 20.0] | 16.0  [12.0, 20.0] | 16.5  [14.0, 19.0] | 19.0  [15.0, 24.5] | 14.0  [14.0, 16.0] | 16.0  [14.0, 20.3] |
| (Min,Max) | (6.0,44.0) | (6.0,44.0) | (11.0,27.0) | (10.0,26.0) | (10.0,18.0) | (10.0,26.0) |
| **Ravu-level** (μg/ml), median [IQR] | 303.0  [221.0, 417.0] | 295.5  [201.5, 411.8] | 375.0  [237.0, 448.0] | - | - | - |
| (Min,Max) | (111.0,655.0) | (111.0,655.0) | (133.0,517.0) | - | - | - |
| **Ravu-level**  **< 175 μg/ml,** n (%) | 11 (19.3%) | 10 (22.7%) | 1 (7.7%) | - | - | - |
| **Ecu-level (**μg/ml), median [IQR] | - | - | - | 299.0  [168.0, 374.0] | 424.5  [247.3, 562.8] | 300.0  [168.0, 378.0] |
| (Min,Max) | - | - | - | (36.0,796.0) | (89.0,604.0) | (36.0,796.0) |
| **Ecu-level**  **< 99 μg/ml,** n (%) | - | - | - | 2 (15.4%) | 1 (25.0%) | 3 (17.6%) |
| abbreviations: AH50: alternative complement pathway activity; CH50: classical complement pathway activity; CI: Confidence interval; Ecu: eculizumab; IQR: inter-quartile range; lab: laboratory assessment(s); Max: maximum; Min: minimum; Ravu: ravulizumab | | | | | | |

**Supplementary Table 3.**

| **Supplementary Table 3. Clinical Scores at baseline and follow-up for patients who continued versus discontinued ravulizumab therapy** | | | |
| --- | --- | --- | --- |
|  | **Overall**  (N=57) | **Ravulizumab continued** (N=39) | **Ravulizumab discontinued** (N=18) |
| **MG-ADL at BL**, median [IQR] | 10.0 [7.0, 12.0] | 9.0 [6.0, 10.3] | 11.5 [9.8, 12.3] |
| missing | 5 | 3 | 2 |
| **MG-ADL at FU**, median [IQR] | 9.0 [7.0, 11.0] | 9.0 [5.0, 10.0] | 11.0 [8.0, 13.0] |
| missing | 3 | 1 | 2 |
| **QMG at BL**, median [IQR] | 16.0 [13.0, 20.0] | 15.0 [12.0, 18.5] | 18.5 [16.5, 22.5] |
| missing | 7 | 3 | 4 |
| **QMG at FU**, median [IQR] | 17.0 [15.0, 21.0] | 17.0 [14.5, 20.0] | 19.0 [16.3, 24.3] |
| missing | 8 | 4 | 4 |
| **QoL15r at BL,** median [IQR] | 20.0 [16.3, 23.0] | 19.5 [16.0, 23.0] | 22.0 [18.0, 23.5] |
| missing | 11 | 5 | 6 |
| **QoL15r at FU**, median [IQR] | 18.0 [14.5, 24.0] | 18.0 [14.0, 21.0] | 24.5 [18.3, 26.3] |
| missing | 10 | 4 | 6 |
| Abbreviations: BL: baseline; FU: follow-up; MG-ADL: Myasthenia gravis Activities of daily living; MG-QoL15r: Myasthenia gravis Quality of Life Score, 15 items, revised version; QMG: Quantitative Myasthenia gravis Score | | | |

**Supplementary Table 4.**

| **Supplementary Table 4. Patient-reported subjective duration of ravulizumab effect** | | | |
| --- | --- | --- | --- |
|  | Overall  (N=57) | Ravulizumab  continued (N=39) | Ravulizumab discontinued (N=18) |
| Subjective duration of  effectiveness^a^, n (%) |  |  |  |
| 0 weeks | 10 (17.5%) | 1 (2.6%) | 9 (50.0%) |
| 2-5 weeks | 11 (19.3%) | 4 (10.3%) | 7 (38.9%) |
| 6-6.5 weeks | 11 (19.3%) | 10 (25.6%) | 1 (5.6%) |
| 7-7.5 weeks | 9 (15.8%) | 9 (23.1%) | 0 (0%) |
| 8 weeks | 11 (19.3%) | 11 (28.2%) | 0 (0%) |
| Missing answer | 5 (8.8%) | 4 (10.3%) | 1 (5.6%) |
| ^a^Answer to the question: “How many weeks does the ravulizumab effect subjectively last?” | | | |

**Figure Legends for Supplementary figures**

**Supplementary figure 1. Overview of timepoints and number of sampling per patient relative to the start of ravulizumab therapy.** Start of ravulizumab therapy or being offered to start ravulizumab therapy is timepoint zero. Data cutoff for all patients was April, 30^th^ 2024. Blue line represents duration of eculizumab therapy. Red line represents duration of ravulizumab therapy. Grey line represents duration of other add-on-therapy (FcRn or IVIG). Dashed grey line marks observation with no add-on-therapy. + marks the timepoint of a blood sample.

**Supplementary figure 2A-D. Correlation of CH50 and AH50 with eculizumab (A,C,blue) and ravulizumab (B,D,red) serum level.** A) and C) Blue dots represent lab assessments under eculizumab therapy. B) and D) Red dots represent lab assessments under ravulizumab therapy.

**Supplementary figure 3A-E. Scatter plots of CH50, AH50, C3d, sC5b9 and C5-inhibitor levels over time since last infusion (weeks) for patients under eculizumab versus ravulizumab therapy.**

For eculizumab dark blue dots represent measurements between 12 and 16 days after last infusion. Lighter blue dots represent shorter intervals (< 12 days after last infusion) or longer intervals (> 16 days after last infusion). For ravulizumab dark red dots represent measurements between 7 to 9 weeks after last infusion. Lighter red dots represent shorter intervals (< 7 weeks after last infusion) or longer intervals (> 9 weeks after last infusion).

**Supplementary figure 4A-B. Comparison of CH50 and AH50 levels under eculizumab and ravulizumab in 13 individual patients with lab assessments under both eculizumab and ravulizumab.** Blue dots represent lab assessments under eculizumab therapy and red dots represent lab assessments under ravulizumab therapy. The dashed vertical lines indicate the infusion of C5-inhibitor prior to the respective lab assessment. Infusions of C5-inhibitor with no lab assessment are left out. All patients were under continued C5-inhibitor therapy as presented in supplementary figure 1.

**Supplementary figure 5A-E.** Scatter plots of CH50, AH50, C3d, sC5b9 and ravulizumab levels over time since last infusion (weeks) for patients who continued (green) versus discontinued (pink) ravulizumab therapy.
