# Supplementary figures and images for: Inhibition of Classical and Alternative Complement Pathway by Ravulizumab and Eculizumab
Source: Ann Clin Transl Neurol. 2025 Nov 19;13(4):688–99. doi: 10.1002/acn3.70251 (PMC13071113; doi:10.1002/acn3.70251)

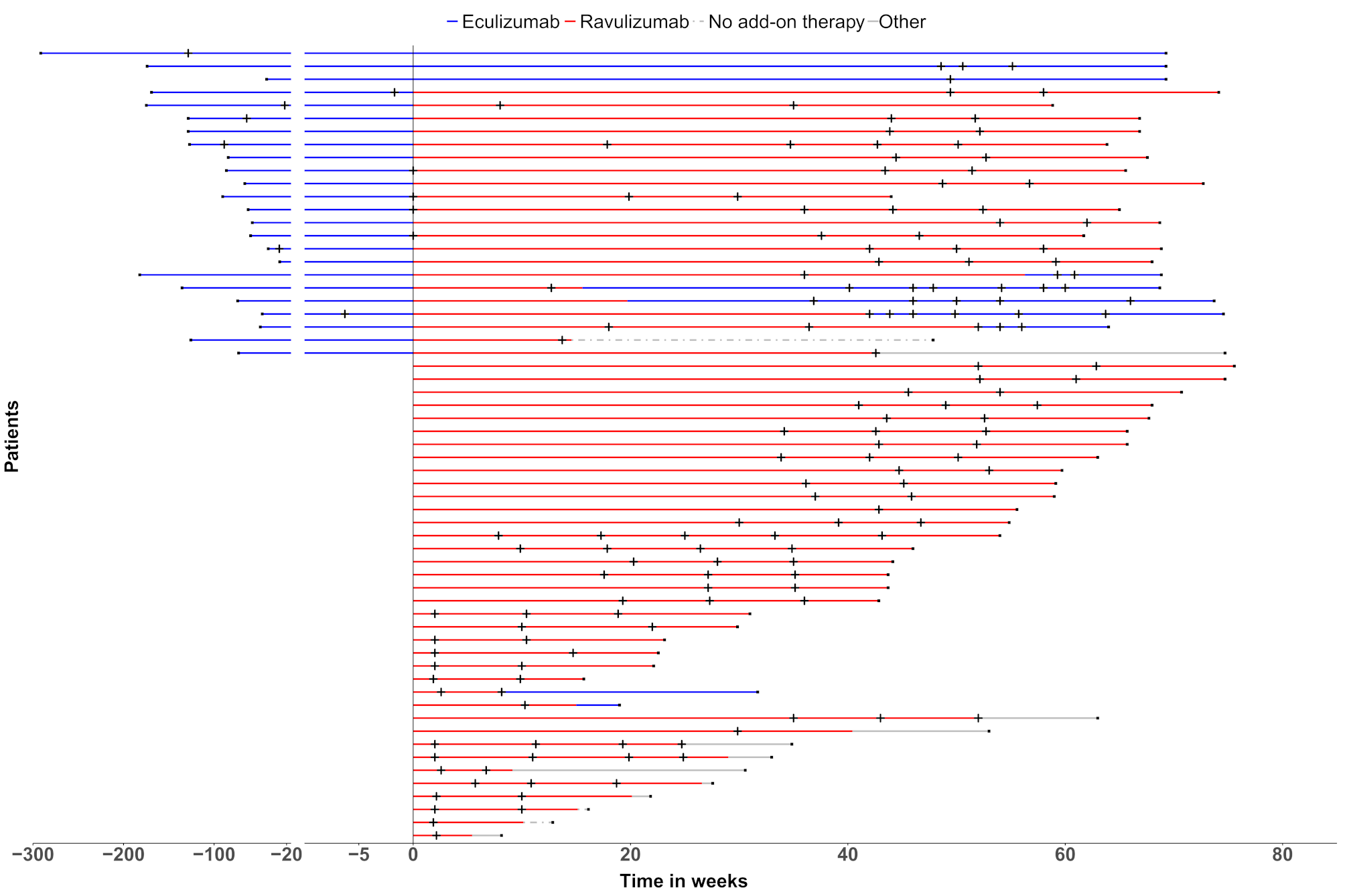

Supplement: Supplementary file 2 — Figure S1: acn370251‐sup‐0002‐FigureS1.tif. [file ACN3-13-688-s006.tif]

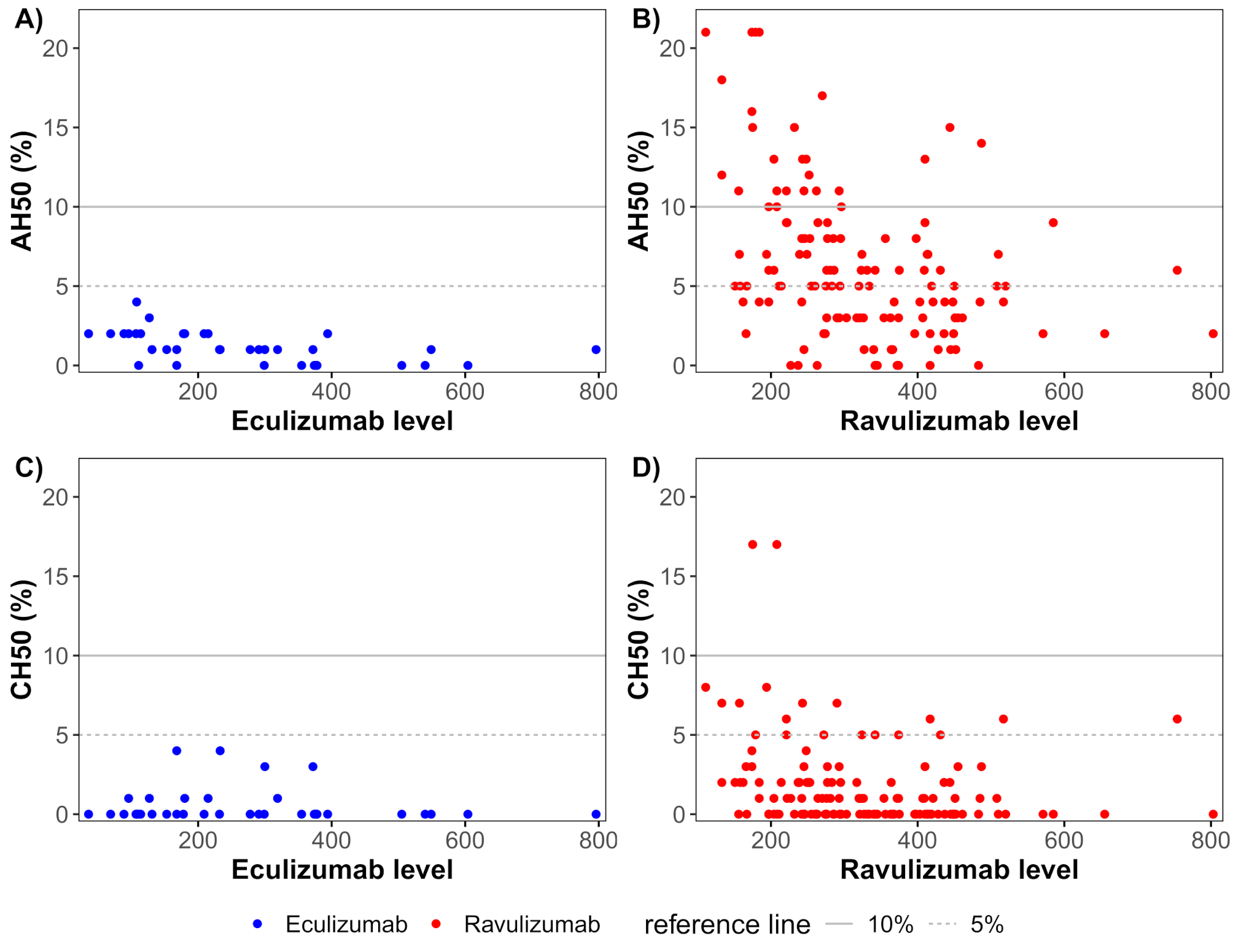

Supplement: Supplementary file 3 — Figure S2: acn370251‐sup‐0003‐FigureS2.tif. [file ACN3-13-688-s003.tif]

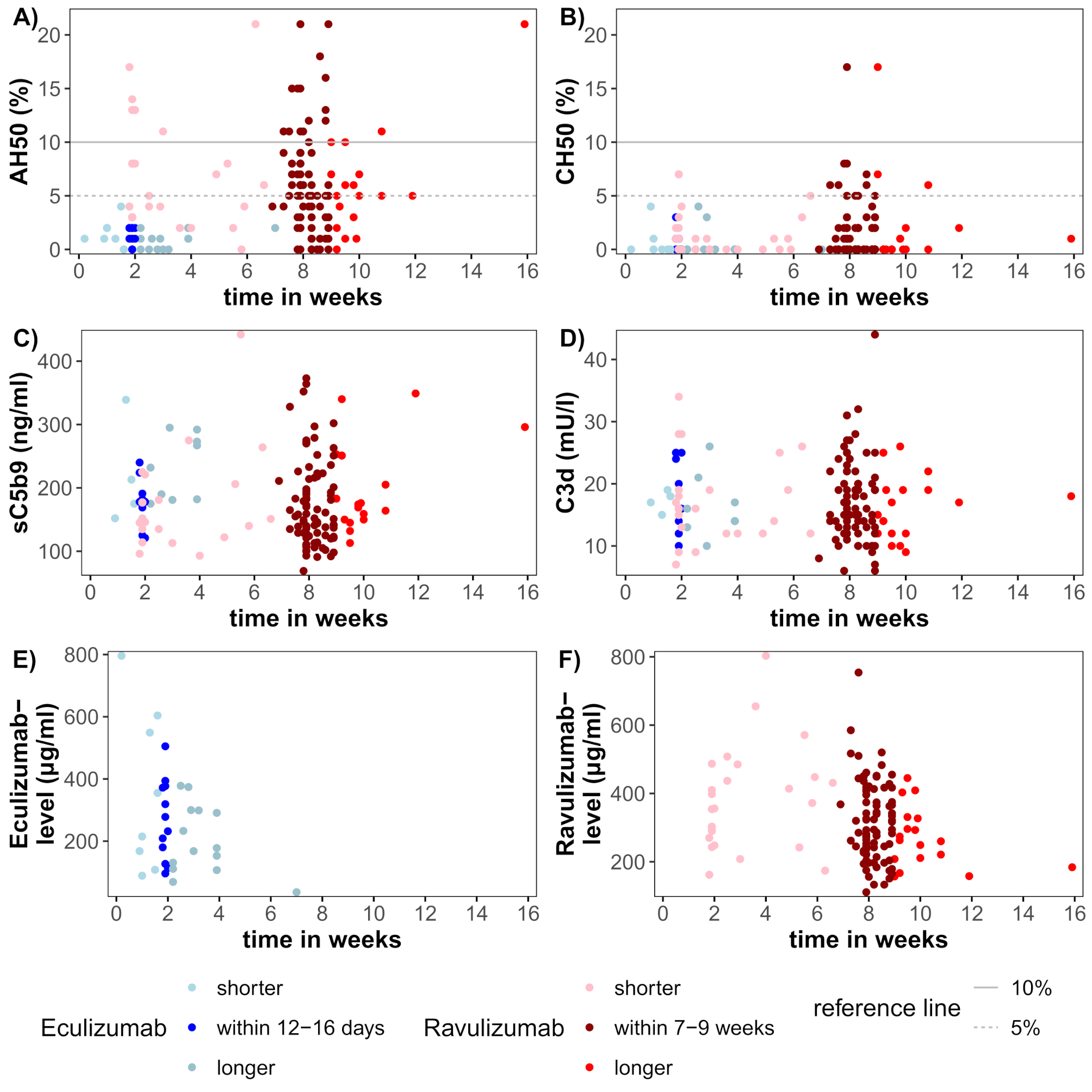

Supplement: Supplementary file 4 — Figure S3: acn370251‐sup‐0004‐FigureS3.tif. [file ACN3-13-688-s001.tif]

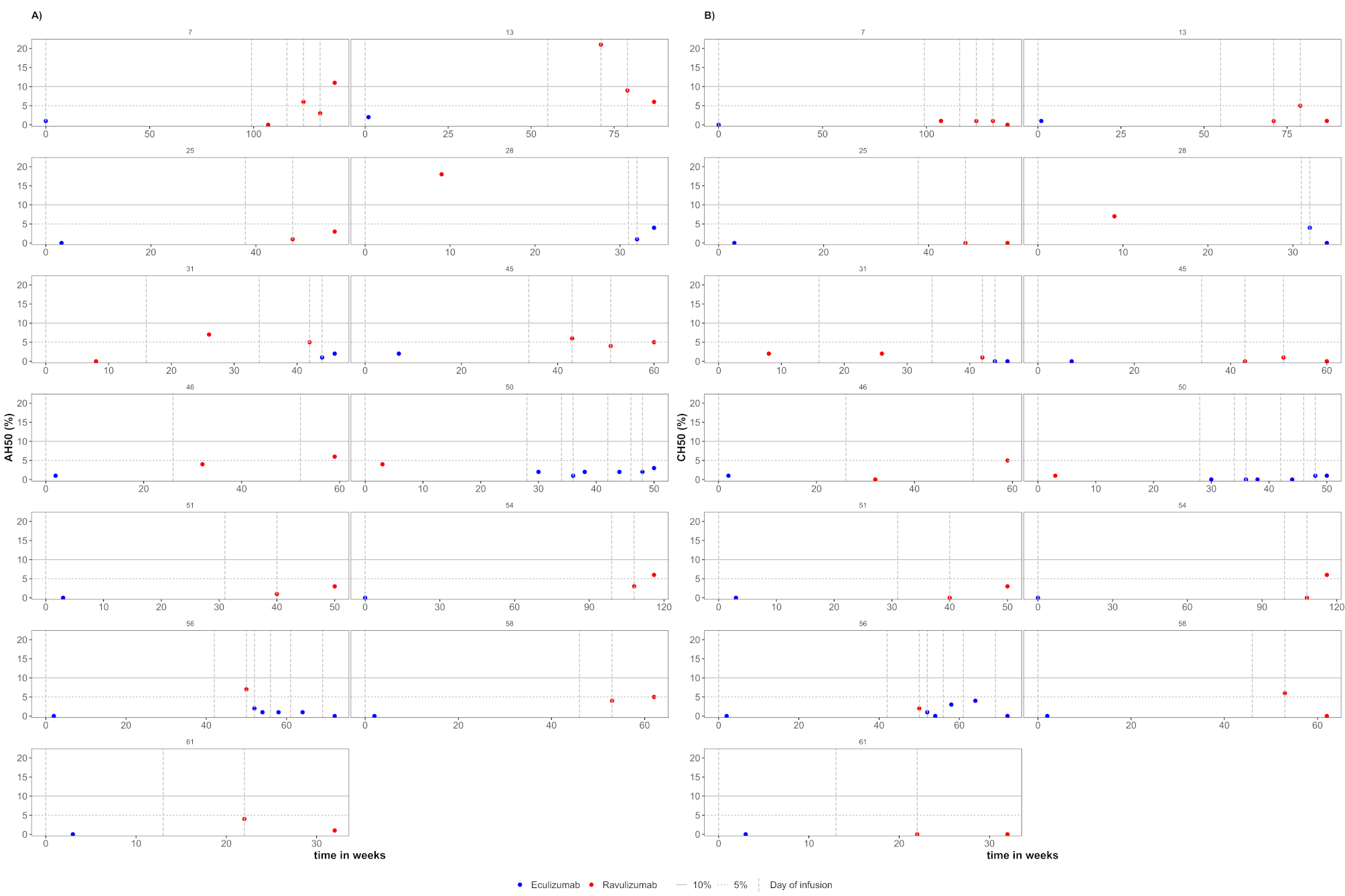

Supplement: Supplementary file 5 — Figure S4: acn370251‐sup‐0005‐FigureS4.tif. [file ACN3-13-688-s002.tif]

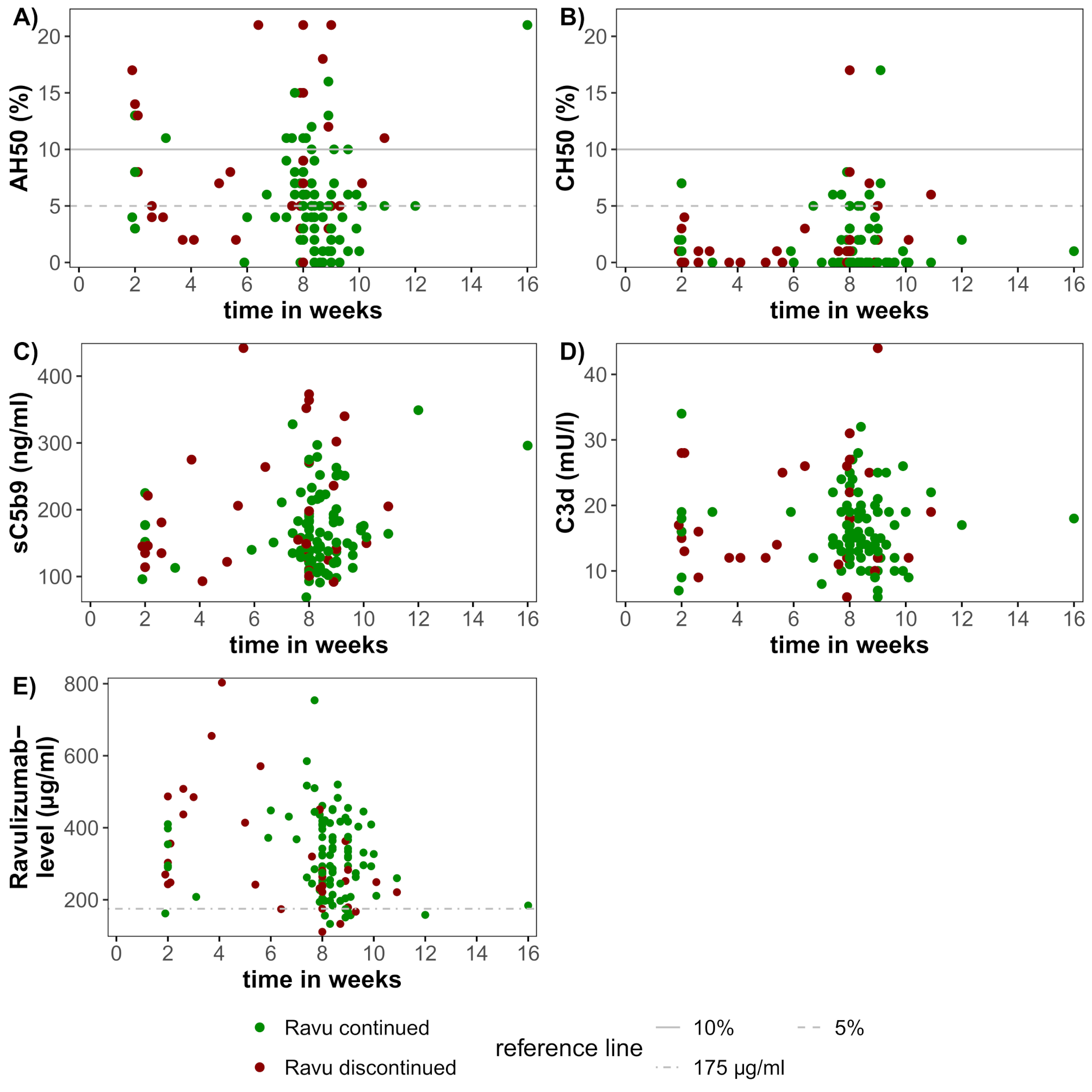

Supplement: Supplementary file 6 — Figure S5: acn370251‐sup‐0006‐FigureS5.tif. [file ACN3-13-688-s005.tif]
